# Molecular characterization of canine circovirus based on the *Capsid* gene in Thailand

**DOI:** 10.1186/s12917-024-04120-w

**Published:** 2024-07-13

**Authors:** Wichan Dankaona, Pornpiroon Nooroong, Napassorn Poolsawat, Nitipon Srionrod, Somporn Techangamsuwan, Panat Anuracpreeda

**Affiliations:** 1 Department of Pathology, Faculty of Veterinary Science, Chulalongkorn University, Bangkok, 10330 Thailand; 2 Animal Virome and Diagnostic Development Research Unit, Faculty of Veterinary Science, Chulalongkorn University, Bangkok, 10330 Thailand; 3 Parasitology Research Laboratory (PRL), Institute of Molecular Biosciences, Mahidol University, Nakhon Pathom, 73170 Thailand

**Keywords:** Canine circovirus, Capsid, Epitope, Evolution, Genetic characterization

## Abstract

**Background:**

Canine circovirus (CanineCV) is a single-stranded circular DNA virus that infects domestic and wild canids in many countries. CanineCV is associated with gastroenteritis and diarrhea, respiratory disease, and generalized vasculitis leading to a fatal event. The Capsid protein (Cap) is a structural protein of the virus which has high genetic variability and plays a role in the canine immune response. In this study, we cloned the full-length CanineCV Capsid gene (*Cap*). In-silico analyses were used to explore the genomic and amino acid variability and natural selection acting on the *Cap* gene. The immune relevance for T-cell and B-cell epitopes was predicted by the immunoinformatic approach.

**Results:**

According to the *Cap* gene, our results showed that CanineCV was separated into five phylogenetic groups. The obtained CanineCV strain from this study was grouped with the previously discovered Thai strain (MG737385), as supported by a haplotype network. Entropy analyses revealed high nucleotide and amino acid variability of the Capsid region. Selection pressure analysis revealed four codons at positions 24, 50, 103, and 111 in the Cap protein evolved under diversifying selection. Prediction of B-cell epitopes exhibited four consensus sequences based on physiochemical properties, and eleven peptide sequences were predicted as T-cell epitopes. In addition, the positive selection sites were located within T-cell and B-cell epitopes, suggesting the role of the host immune system as a driving force in virus evolution.

**Conclusions:**

Our study provides knowledge of CanineCV genetic diversity, virus evolution, and potential epitopes for host cell immune response.

**Supplementary Information:**

The online version contains supplementary material available at 10.1186/s12917-024-04120-w.

## Background

Canine circovirus (CanineCV) comprises a small, non-enveloped, covalently closed circular, single-stranded DNA (ssDNA) genome. It belongs to the genus *Circovirus* in the family *Circoviridae.* The DNA genome of CanineCV consists of ∼ 2 kb of nucleotides that contains two major open reading frames (ORFs) arranged in the opposite orientation [1]. ORF1 or the replicase (*Rep*) gene encodes 303 amino acids of a replicase-associated (Rep) protein, while ORF2, or the capsid (*Cap*) gene, encodes 270 amino acids of a viral capsid (Cap) protein. CanineCV was first discovered in dog sera in the USA in 2012 [1]. It has been reported that CanineCV is associated with hemorrhagic gastroenteritis, diarrhea [2–8], and respiratory problems [9–11]. Notably, a fatal outcome occurred in dogs presented with severe hemorrhagic diarrhea, systemic vasculitis, and hemorrhage [7, 12, 13].

Up to now, the genetic variability of this virus has been continuously demonstrated and revealed that CanineCV has been segregated into six genotypes [14]. Genetic recombination has been identified for CanineCV evolution [8, 10, 11]. Nevertheless, the evolutionary characteristic of the CanineCV, especially the *Cap* gene under natural selection has not been well defined. With respect to the Cap protein, this protein is a key structural protein that has been studied since it can stimulate a strong immunological response compared to the Rep protein [15]. In CanineCV, seroconversion of specific antibodies to the recombinant CanineCV Cap protein was identified [16]. It was found that antigenic epitopes which could interact with T-cell associated molecules were located on the Cap protein [17]. However, the study of antigenic T-cell epitopes is still limited, and the B-cell epitopes of CanineCV have not been demonstrated. This study, therefore, aimed to investigate the genetic diversity of the CanineCV *Cap* gene derived from oronasal swabs of a CanineCV-infected dog showing respiratory disease. An evolution of the CanineCV *Cap* gene under natural selection was identified. Additionally, immune-relevant B-cell and T-cell epitopes were also proposed.

## Results

### Construction of recombinant CanineCV Cap gene

The amplified full-length sequence of the CanineCV *Cap* gene from the Thailand strain was 813 bp, encoding 270 amino acids for the Cap protein. The nucleotide sequence has been deposited in the GenBank database with accession number ON863358.

### Phylogenetic and similarity analysis of the CanineCV Cap gene

**Fig. 1:**
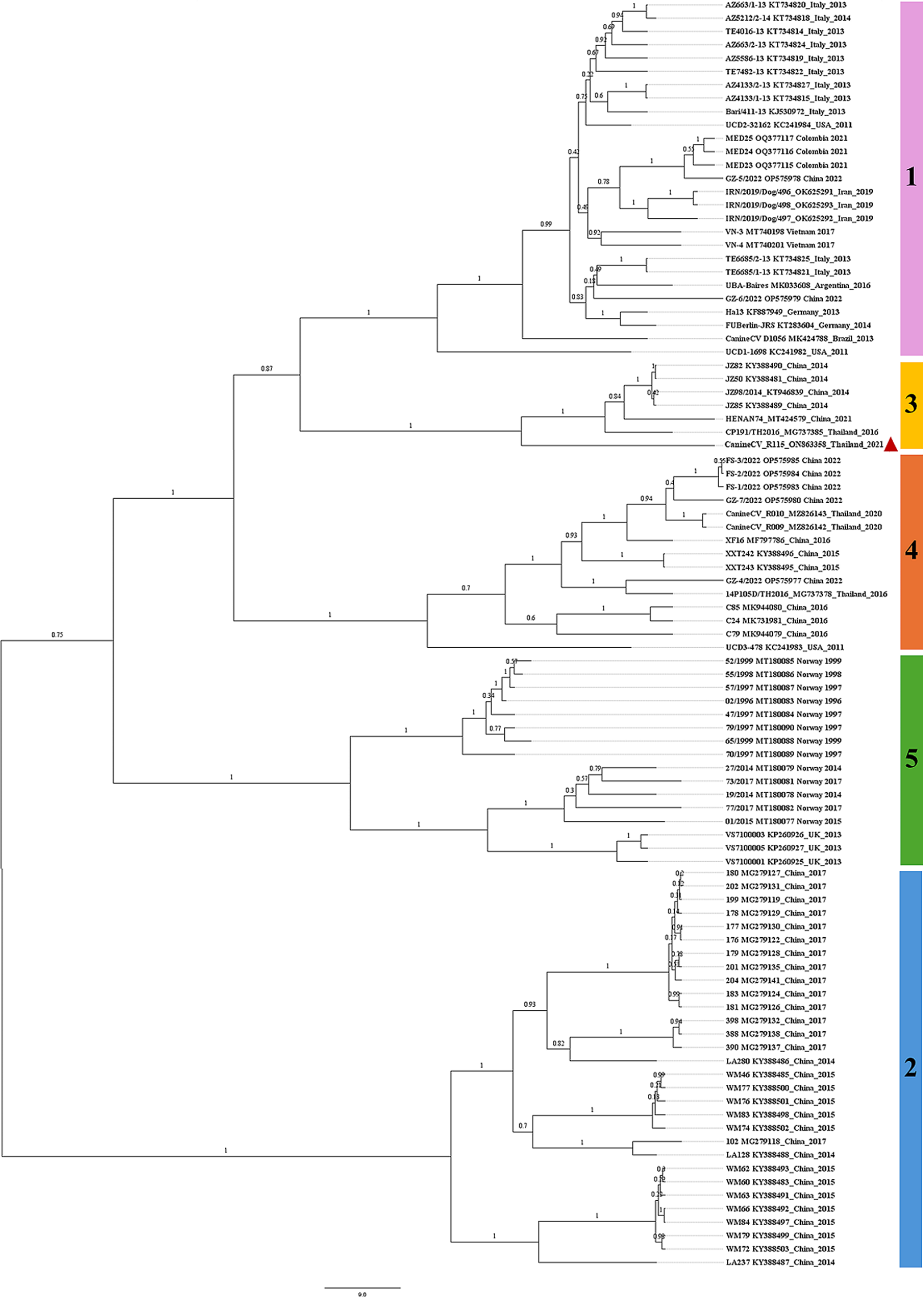
A Bayesian phylogenetic cladogram of CanineCV *Cap* sequence in this study (red triangle) and those taken from GenBank. The reliability of the phylogeny was evaluated using posterior probability branch support values. The scale bar was scaled to the range of the branch lengths (in units of year) of the phylogeny

The Bayesian phylogenetic analysis, based on the alignment of CanineCV *Cap* gene sequences obtained in this work with other ninety-four sequences retrieved from the GenBank, including those from the USA, UK, Norway, Brazil, Germany, Argentina, Italy, Iran, Vietnam, Colombia, China, and Thailand, were classified into five clades (designated as clade 1, 2, 3, 4, and 5). Our sequence from this study was positioned in clade 3 with other sequences from Thailand and China. The 1st clade consisted of CanineCV sequences from the USA, Brazil, Germany, Argentina, Italy, Iran, Colombia, and Vietnam, while the 2nd clade contained the sequences from China. The 4th clade comprised CanineCV sequences from the USA, Thailand and China. In addition, CanineCV sequences from the UK and Norway were grouped within the 5th clade (Fig. 1). The reliability of the phylogeny was shown with a posterior probability value of the branch support. Additionally, the similarity among Thai strains ranged from 86.22 to 99.88%, as demonstrated in Table 1. The percentage of nucleotide sequence similarity of the *Cap* gene within each clade ranged from 89.54 to 100.00% (1st clade), 77.24–100.00% (2nd clade), 86.59–99.88% (3rd clade), 84.13–100.00% (4th clade), and 81.06–99.75% (5th clade), as provided in Supplementary Table 1. For the nucleic acid substitution pattern, our findings showed that the rate of transitional substitution was greater than that of the transversional substitution (Table 2), with an overall relative percentage of Adenine (A), Thymine (T), Cytosine (C), and Guanine (G) at 31.80, 21.00, 25.50, and 21.70, respectively.

**Table 1:** Similarity of the CanineCV *Cap* sequences as examined in dog samples in Thailand

Clade	3		4		
Accession no.	1	2	3	4	5
1. **ON863358**					
2. MG737385	94.59				
3. MZ826142	88.68	86.22			
4. MZ826143	88.68	86.22	99.88		
5. MG737378	88.68	87.21	96.06	96.06	

The accession number for *Cap* gene obtained in this study was shown in boldface

**Table 2:** The nucleic acid substitution rate and base composition of CanineCV *Cap* gene. Each entry is the probability of substitution from one base (row) to another base (column). Rates of different translational substitutions are shown in boldface and those of transversional substitutions are shown in italics. The maximum Log likelihood for this analysis was − 8524.095

From\To	A	T	C	G
A	-	*5.19*	*5.19*	**14.62**
T	*5.19*	-	**14.62**	*5.19*
C	*5.19*	**14.62**	-	*5.19*
G	**14.62**	*5.19*	*5.19*	-

### Haplotype diversity

**Fig. 2:**
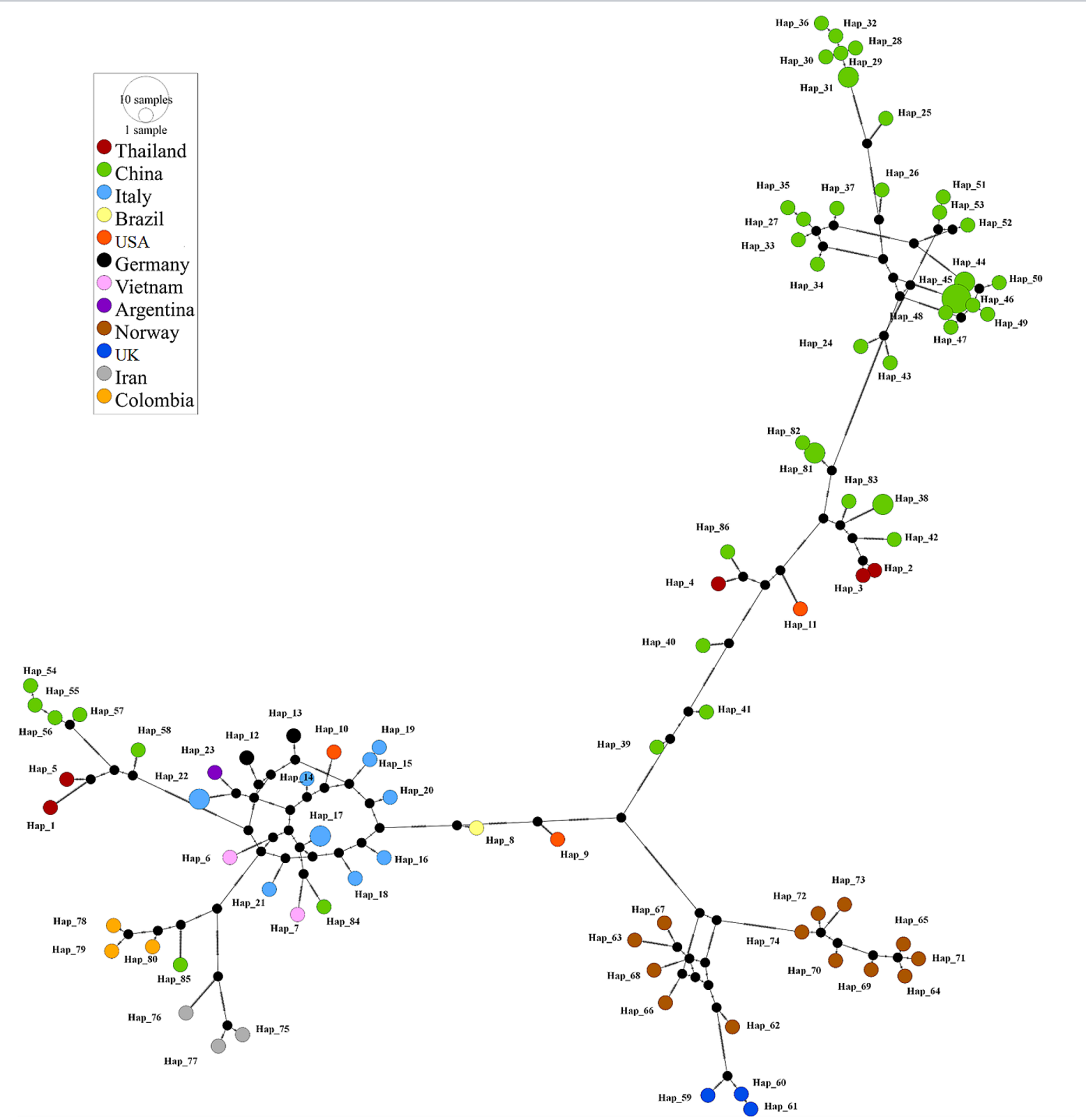
TCS network of haplotypes based on CanineCV *Cap* sequences detected in Thailand and worldwide countries. The size of the circle is proportional to the size of each haplotype which is color coded according to the identified country. The slash mark between each haplotype represents the one base pair difference. The black circles are the intermediate traits caused by the single nucleotide polymorphism (SNP)

The haplotype analysis showed that all retrieved CanineCV *Cap* sequences were assigned into 86 haplotypes. In haplotype #17, CanineCV strains AZ4133/1–13 (KT734815) and AZ4133/2–13 (KT734827) from Italy were grouped together. Similarly, CanineCV strains TE6685/1–13 (KT734821) and TE6685/2–13 (KT734825) from Italy were grouped in haplotype #22. CanineCV strain WM66 (KY388492) and WM84 (KY388497) from China formed haplotype #31, while strains XXT242 (KY388496) and XXT243 (KY388495) from China were in haplotype #38. CanineCV strain 176 (MG279122) and 177 (MG279130) from China were grouped together in haplotype #44. Strain 178 (MG279129), 180 (MG279127), 199 (MG279119), and 202 (MG279131) from China were grouped in haplotype #45. Finally, strains FS-2/2022 (OP575984) and FS-3/2022 (OP575985) from China were in haplotype #81. The TCS network illustrated that the CanineCV *Cap* sequence from this study, defined as haplotype #1, was closest to haplotype #5, which is the CP191/TH2016 (MG737385) strain, another Thai sequence, with 43 mutational occurrences. Other Thai sequences, including CanineCV_R009 (MZ826142), CanineCV_R010 (MZ826143), and 14P105D/TH2016 (MG737378), were found in haplotype #2, #3, and #4, respectively. The remaining haplotypes were from other countries worldwide, as demonstrated in Fig. 2. The values for haplotype diversity (Dh), nucleotide diversity (π), and the average number of nucleotide differences (*K*) were 1.000 ± 0.002, 0.328 ± 0.007 and 119.566, respectively.

### Entropy analysis

**Fig. 3:**
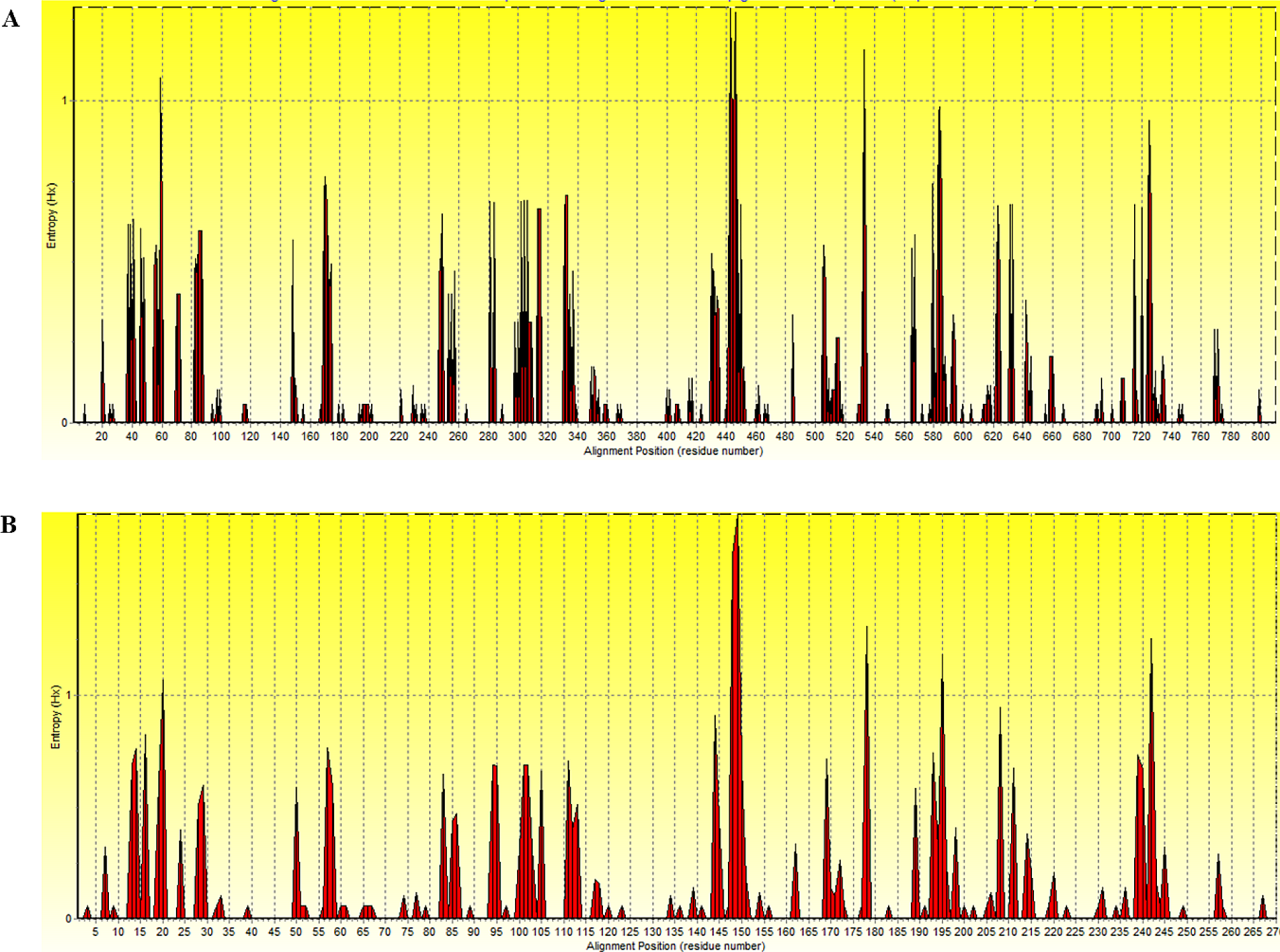
Entropy plot of multiple nucleic acid (**A**) and amino acid (**B**) sequence alignment of the CanineCV *Cap* gene and protein, respectively. The red peaks indicate the high variation at each position of nucleic (A) and amino (B) acid sequences

Entropy analysis showed high variation along the *Cap* nucleotide sequence alignment, with 211 peaks with the highest entropy value of 1.291 at nucleotide position 443 (Fig. 3A). The entropy of amino acid sequences exhibited in the chart showed 101 entropy peaks with the highest entropy value of 1.812 at amino acid position 149 (Fig. 3B).

### Prediction of nuclear localization signal (NLS)

The CanineCV Cap protein obtained from this study consisted of 270 amino acids. The predicted NLS sequence was ^11^RRRYRTRPLIRYRRRRQNNFKMFHLRLRRT^40^, which was located at the N-terminus of the Cap protein. An arginine (R) was identified as the most frequent amino acid residue (43.33%, 13/30) within the NLS region.

### Population dynamics

**Fig. 4:**
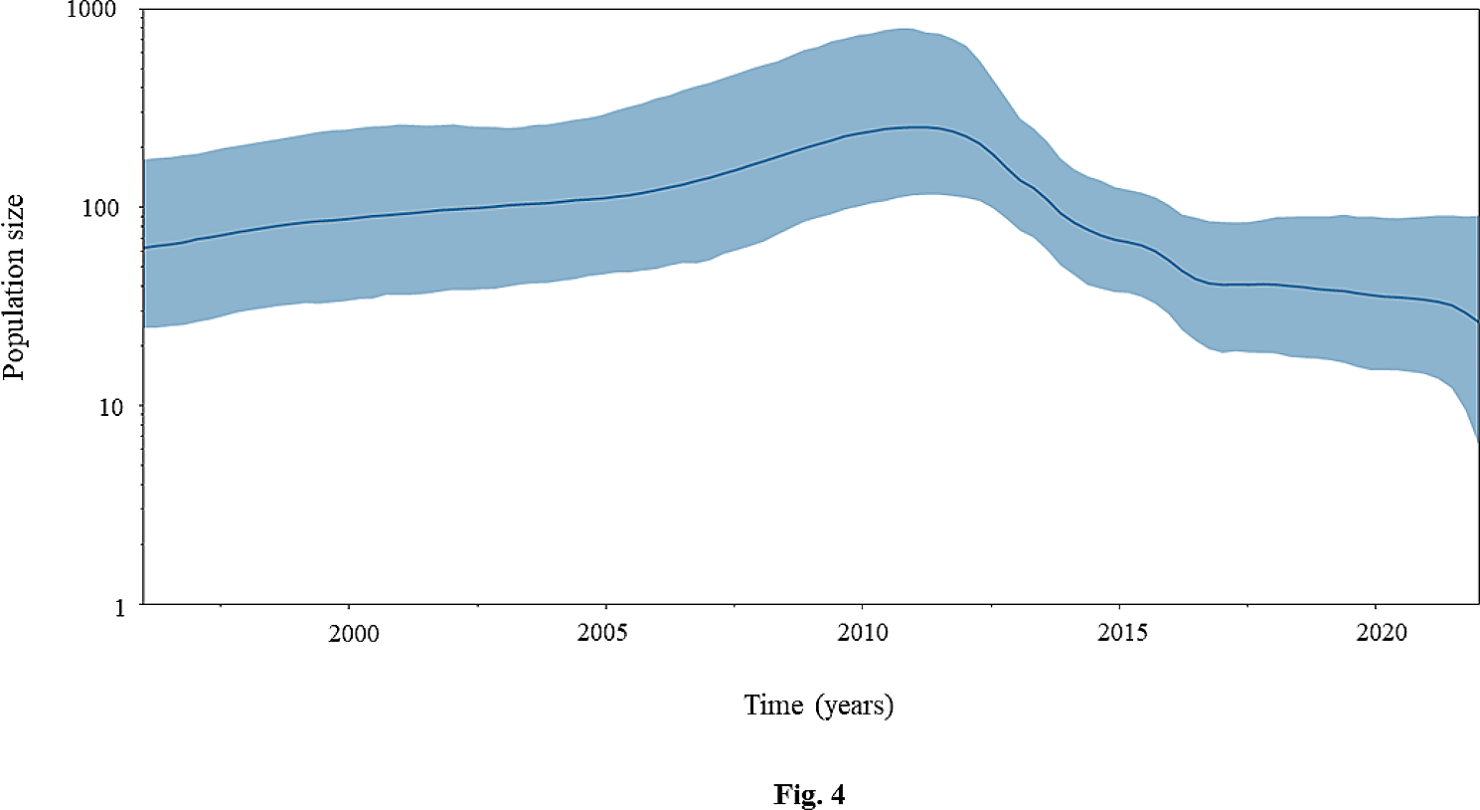
Bayesian Skyride plot showing the posterior median (solid blue line) and 95% Bayesian credible intervals (BCIs) (blue shading) based on the CanineCV *Cap* gene. The y-axis depicts an effective population size as a log transformation of population size, while the x-axis indicates the time from past to present

According to the Bayesian Skyride plot, the effective population size of CanineCV slowly increased from 1996, when the oldest strains were discovered, reaching its peak around 2012. Subsequently, from 2012 to 2016, the effective population size gradually decreased, followed by a slight decline until the discovery of the youngest strains in 2022 (Fig. 4).

### Selection pressure

Datamonkey analysis revealed that there were 10, 4, and 6 sites indicating statistically significant purifying selection according to FEL, SLAC, and FUBAR, respectively (Supplementary Table 2). Conversely, significant diversifying selection was observed at 4, 1, 4, and 45 sites by FEL, SLAC, FUBAR, and MEME, respectively (Table 3). Among these, 4 codon positions, specifically 24, 103, 111, were detected by 3 methods (FEL, FUBAR, MEME), with only codon position 50 identified as under diversifying selection by all methods. Additionally, the mean dN/dS value for the entire *Cap* gene derived from the SLAC was 0.729.

**Table 3:** Codon positions under diversifying selection

Codon position		FEL		SLAC		FUBAR		MEME	
		dN/dS	*p*-value	dN/dS	*p*-value	dN/dS	Prob (dN > dS)	dN/neutral evolution component	*p*-value
19	1.256		0.827	1.213	0.600	0.829	0.387	**238.42**	**0.000**
24	**Infinity**		**0.037**	2.189	0.202	**7.467**	**0.915**	**584.250**	**0.000**
28	0.420		0.495	0.550	0.884	0.596	0.417	**1719.740**	**0.010**
29	3.990		0.310	0.321	0.948	0.440	0.329	**8693.050**	**0.000**
39	0.000		0.327	2.469	0.522	1.324	0.66	**238.250**	**0.000**
50	**Infinity**		**0.055**	**9.043**	**0.069**	**7.855**	**0.950**	**25.220**	**0.070**
57	0.434		0.571	1.382	0.612	0.657	0.401	**1795.310**	**0.000**
58	0.565		0.471	0.704	0.823	0.711	0.065	**6106.950**	**0.000**
65	0.082		0.112	0.082	0.994	0.239	0.196	**24658.290**	**0.010**
66	0.160		0.195	0.215	0.974	0.262	0.265	**17485.640**	**0.000**
77	Infinity		0.317	3.440	0.422	2.076	0.737	**160.780**	**0.020**
83	0.482		0.384	0.704	0.800	0.484	0.103	**78563.810**	**0.000**
100	0.824		0.891	1.450	0.487	1.090	0.494	**37692.940**	**0.000**
102	0.206		0.015	0.771	0.790	0.240	0.019	**61548.010**	**0.000**
103	**Infinity**		**0.020**	2.308	0.132	**16.355**	**0.968**	**688.490**	**0.000**
105	Infinity		0.134	1.088	0.634	1.800	0.69	**2342.400**	**0.000**
111	**Infinity**		**0.051**	1.261	0.421	**10.813**	**0.971**	**2921.020**	**0.000**
117	0.969		0.987	1.493	0.518	1.236	0.616	**100000.000**	**0.000**
118	Infinity		0.377	2.607	0.346	2.471	0.793	**41.470**	**0.060**
120	Infinity		0.328	2.464	0.523	1.322	0.66	**189.450**	**0.000**
123	23.229		0.655	2.065	0.592	1.302	0.585	**7515.460**	**0.010**
136	Infinity		0.446	2.483	0.521	1.678	0.689	**1692.420**	**0.000**
139	Infinity		0.434	Infinity	0.664	1.443	0.742	**377.070**	**0.000**
144	1.103		0.885	1.451	0.414	0.939	0.176	**710.280**	**0.080**
145	1.973		0.605	2.114	0.368	1.836	0.699	**36105.340**	**0.000**
149	1.363		0.651	1.717	0.318	1.119	0.207	**2149.200**	**0.000**
156	Infinity		0.112	0.082	0.994	0.240	0.196	**15474.400**	**0.020**
171	0.219		0.199	0.323	0.940	0.393	0.254	**1138.890**	**0.000**
172	Infinity		0.166	2.441	0.362	1.973	0.774	**940.120**	**0.000**
177	0.683		0.763	0.993	0.743	0.855	0.454	**3869.740**	**0.000**
178	0.733		0.686	0.850	0.740	0.849	0.105	**1320.070**	**0.000**
194	0.262		0.382	1.012	0.734	0.489	0.421	**18394.290**	**0.000**
195	0.765		0.696	1.070	0.588	0.827	0.076	**4307.630**	**0.000**
196	0.469		0.512	0.571	0.858	0.642	0.308	**480.140**	**0.000**
198	0.263		0.078	0.350	0.967	0.382	0.012	**1320.410**	**0.000**
205	0.772		0.839	0.991	0.744	0.948	0.476	**4532.940**	**0.000**
206	0.540		0.711	1.154	0.630	0.632	0.396	**9150.990**	**0.000**
208	Infinity		0.156	Infinity	0.254	3.048	0.879	**23.470**	**0.090**
211	0.466		0.536	1.340	0.493	0.911	0.367	**16887.420**	**0.000**
220	0.850		0.879	1.284	0.576	0.974	0.491	**735.490**	**0.000**
230	0.070		0.189	0.549	0.845	0.171	0.323	**47879.560**	**0.010**
236	1.038		0.977	1.245	0.679	1.167	0.566	**561.620**	**0.000**
239	2.987		0.452	2.499	0.406	2.220	0.775	**117.860**	**0.010**
249	0.900		0.967	1.473	0.671	1.024	0.527	**44.930**	**0.090**
257	0.191		0.181	0.931	0.708	0.339	0.22	**138.530**	**0.010**

A significant *p*-value for diversifying selection was shown in boldface

### Epitopes analysis

**Fig. 5:**
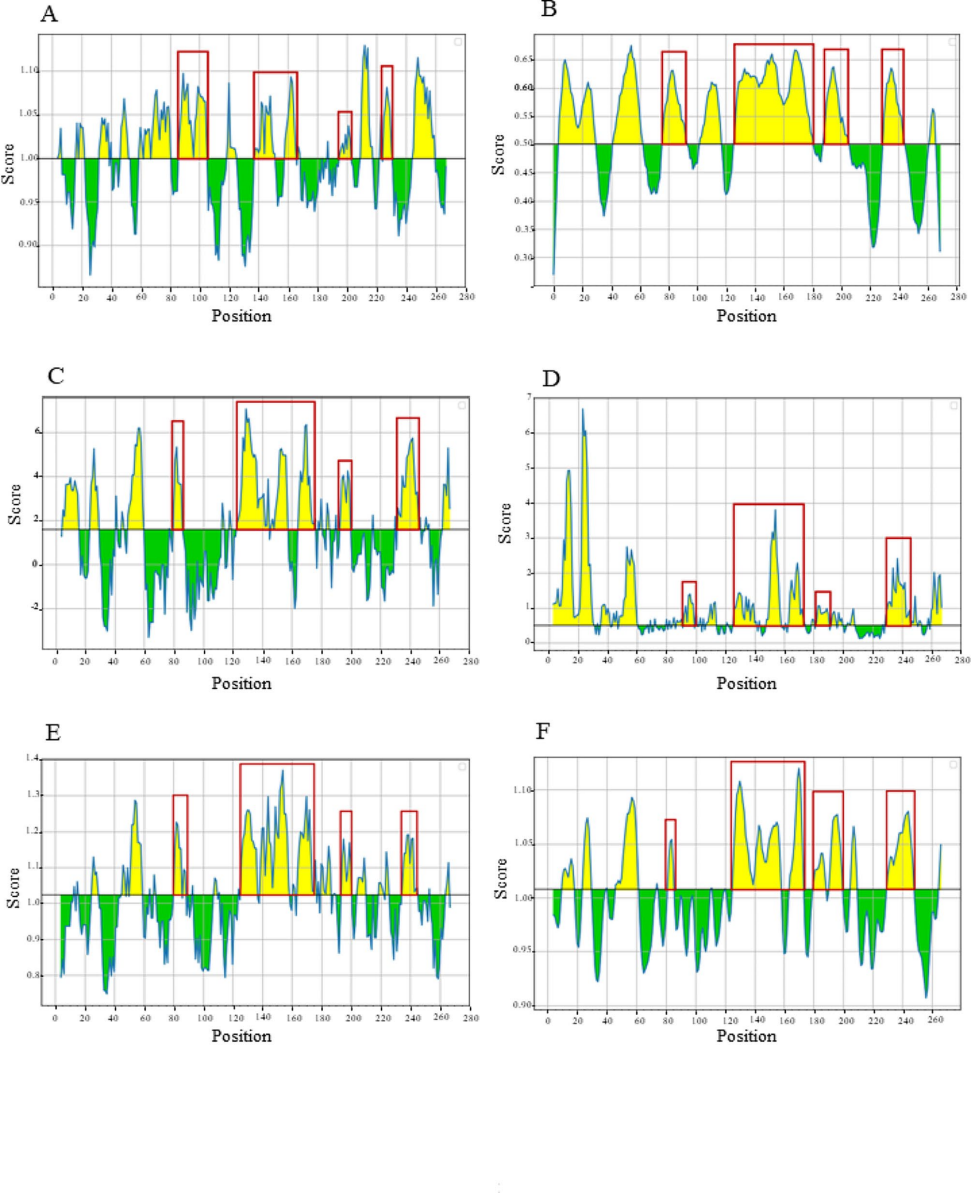
B-cell epitope prediction for CanineCV *Cap* protein using IEDB software. The physicochemical properties of CanineCV *Cap* protein were predicted based on Kolaskar & Tongaonkar antigenicity (**A**), BepiPred linear epitope (**B**), Parker hydrophilicity (**C**), Emini surface accessibility (**D**), Chou & Fasman Beta-turn prediction (**E**) and Karplus & Schulz flexibility prediction (**F**). The X-axis represents the positions in the Cap peptide sequence, while the Y-axis indicates the propensity score for each residue. Yellow color areas depict the probability of being part of the epitope according to the determination threshold value shown in the red line

**Fig. 6:**
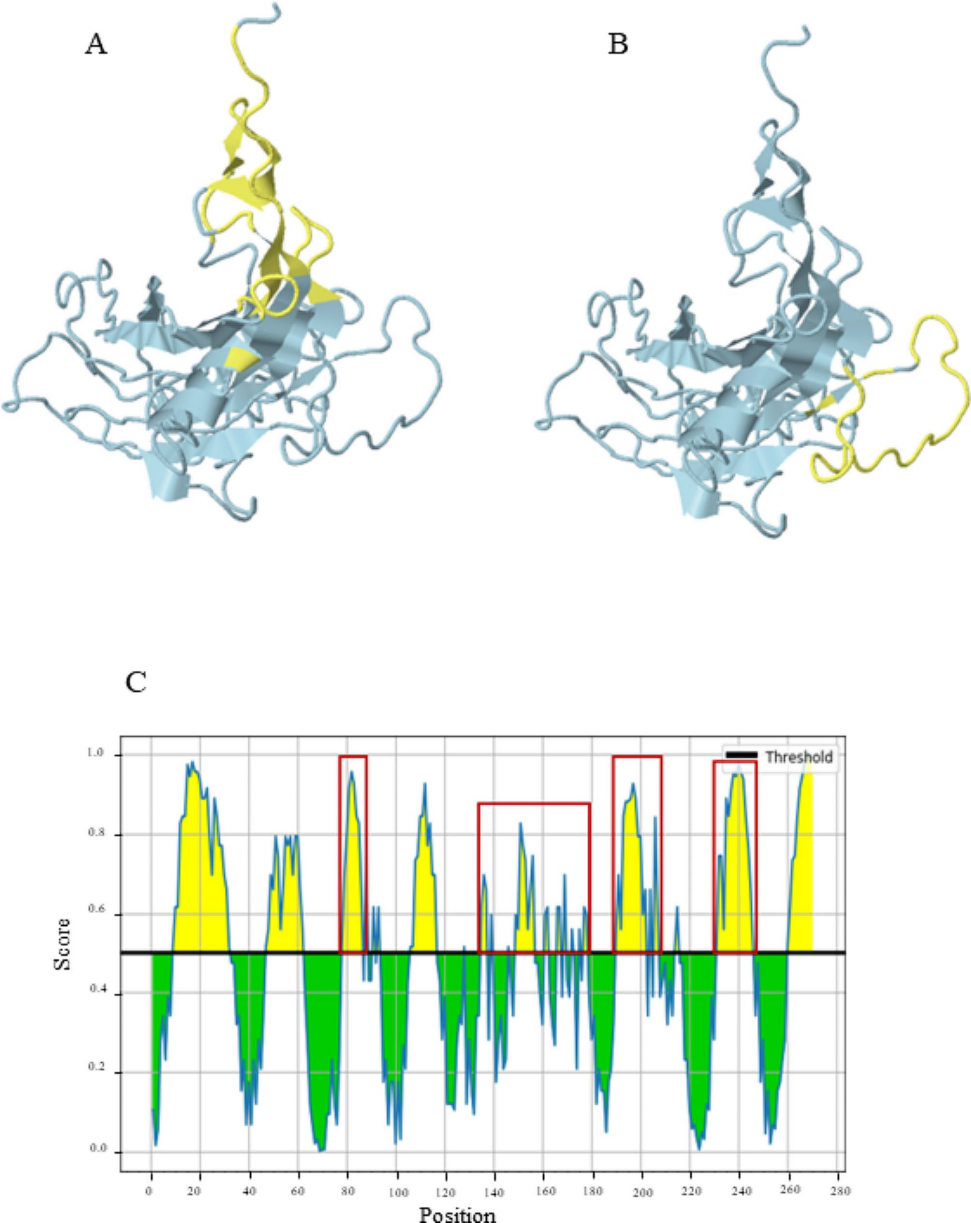
B-cell epitope prediction for CanineCV *Cap* protein using ElliPro server. The predicted discontinuous B-cell epitopes based on 3D structure mapping of the CanineCV Cap protein (**A** and **B**) showed the areas covered by the B-cell epitopes (yellow) that overlapped with the same positions in the linear B-cell structure anticipation (**C**)

B-cell and T-cell epitopes were predicted based on CanineCV Cap peptide sequence. Focusing on continuous B-cell epitopes prediction, there were ten amino acid sequences as potential epitopes which were relatively conserved (Table 4). Regarding the Kolaskar & Tongaonkar antigenicity method, the average antigenicity of the Cap protein was 1.000, with a maximum of 1.129 and a minimum of 0.866 at the antigen determination threshold value of 1.000 (Fig. 5A). To enhance the accuracy of predicting continuous B-cell epitopes, prediction methods including BepiPred linear epitope, Parker hydrophilicity, Emini surface accessibility, Chou & Fasman Beta-turn prediction, and Karplus & Schulz flexibility prediction were used. The BepiPred linear epitope algorithm identified eight peptide regions as potential epitopes (Fig. 5B and Supplementary Table 3). Additionally, the Parker hydrophilicity and Emini surface accessibility prediction tools proposed nine regions with a high hydrophilicity (Fig. 5 C and Supplementary Table 4) and six regions with a high probability of being located on the Cap protein surface (Fig. 5D and Supplementary Table 5), respectively. The Chou & Fasman Beta-turn algorithm detected twelve Beta-turn regions within the sequence (Fig. 5E and Supplementary Table 6), while the Karplus & Schulz flexibility prediction revealed ten flexible regions in the Cap protein (Fig. 5F and Supplementary Table 7). A combination of predicted results from each parameter showed four overlapping amino acid regions (Fig. 5A-F) that could be candidates for B-cell epitope recognition and achievable for antibody binding. In addition to continuous B-cell epitopes, the prediction of discontinuous B-cell epitopes was evaluated based on the 3D structure of the Cap protein by the ElliPro server (Fig. 6A-B; Table 5), which also overlapped with the linear sequence of B-cell epitopes (Fig. 6 C). The yellow color in ElliPro graph indicated the antigenicity properties in that area of Cap protein related with the BepiPred linear epitope prediction.

**Table 4:** Analysis of linear B-cell epitopes predicted from the Cap peptide sequence determined in this study

Number	Start	End	Peptide	Conserved amino acid / Total amino acid
1	32	38	MFHLRLR	5/7
2	68	80	HLSFKLTDFLQAS	9/13
3	86	105	FQHLPPFRFYKFKKIYVRAK	12/20
4	119	125	GRTALDL	5/7
5	138	151	SHLDPGTVPGLSEP	7/14
6	158	165	APFIYDPL	7/8
7	195	202	TSPSATAP	5/8
8	209	217	PWVSVIQGA	6/9
9	225	230	SISLRQ	5/6
10	245	259	QIPQVQYDISAYITF	11/15

**Table 5:** Analysis of discontinuous B-cell epitopes predicted from the 3D structure of the CanineCV Capsid peptide sequence in this study

Epitope number	Residues	Number of residues	Score
1	R23, Q27, N28, N29, F30, K31, L77, Q78, A79, S80, H81, G82, T83, G84, D85, F86, Q87, P90, P91, R93, S212, V213, I214, Q215, G216, A217, N218, K260, E261, F262, D263, Y264, E265, T266, G267, R268	36	0.68
2	K187, M189, T191, Q192, D193, I194, T195, S196, P197, S198, A199, T200, A201, P202, W203, L204, T205, R206, G207, T208, P209	21	0.646

In addition, the T-cell epitopes predicted based on the Cap amino acid sequence were only found in the MHC class I binding site. As shown in Table 6, eleven peptide regions were considered to have strong binding efficacy with high epitope prediction scores. However, only six out of eleven peptides had an antigenicity score greater than 0.5 based on the VaxiJen evaluation. Interestingly, seven out of eleven peptide regions shared consensus peptides with the B-cell epitope sequences.

**Table 6:** Prediction of conserved peptide sequences of CanineCV Cap antigen for precessing and binding to MHC class I of T-cell epitopes

Allele	Start	End	Length	Peptide	Epitope prediction score	Percentile rank	VaxiJen score
DLA-8,850,801	15	23	9	RTRPLIRYR	0.506259	0.22	1.1896^*^
DLA-8,850,801	**31**	**39**	9	KMFHLRLRR	0.586756	0.12	0.778^*^
DLA-8,850,801	**88**	**96**	9	HLPPFRFYK	0.703917	0.04	0.1583
DLA-8,803,401	101	109	9	YVRAKWINW	0.406047	0.25	2.1568^*^
DLA-8,850,801	111	120	10	KTMMENVLGR	0.460722	0.29	-0.6666
DLA-8,850,801	112	120	9	TMMENVLGR	0.615539	0.10	-0.5524
DLA-8,850,801	**160**	**168**	9	FIYDPLQDR	0.682513	0.06	-0.0399
DLA-8,850,101	**212**	**220**	9	SVIQGANMV	0.431024	0.25	0.5916^*^
DLA-8,850,101	**219**	**228**	10	MVWNGLSISL	0.418579	0.26	0.9690^*^
DLA-8,850,801	**249**	**257**	9	VQYDISAYI	0.507105	0.22	0.1127
DLA-8,803,401	**254**	**262**	9	SAYITFKEF	0.373039	0.3	1.0686^*^

The VaxiJen score with cut-off above 0.5 is shown with asterisk. The peptide sequences sharing with B-cell epitopes are shown in boldface

## Discussion

Nowadays, CanineCV is becoming widely studied since the virus causes serious fatal events in domestic and wild carnivores. Although many studies have demonstrated a genetic diversity of CanineCV, the selection pressure affecting CanineCV has not been well established. Moreover, epitopes for T-cell and B-cell on the CanineCV Capsid protein have not been well defined. In this study, we characterized the *Cap* gene of CanineCV constructed by cloning technique. The *Cap* sequences were analyzed for genetic diversity and natural selection pressure. The antigenicity of B-cell and T-cell epitopes was also proposed based on the Cap amino acid sequence.

Phylogenetic analysis of CanineCV *Ca*p gene sequences revealed that the *Cap* sequence from this study was grouped within the 3rd clade, together with sequences from China and previously discovered Thailand sequences. The phylogram result was supported by the haplotype analysis, which showed that our *Cap* Thailand sequence (accession number ON863358), defined as haplotype #1 was closest to another Thailand sequence (accession number MG737385), along with sequences from China. Other Thailand sequences (accession number MZ826142, MZ826143 and MG737378) were grouped in different haplotypes together with sequences from China and the USA. This finding indicated that CanineCV has some genetic diversity of the *Cap* genes observed in different haplotype networks in Thailand and other countries. As well, these *Cap* genes may share genetic traits with sequences determined earlier worldwide. However, our findings are not consistent with a previous publication from Iran reported by Beikpour et al. (2022), who described CanineCV being divided into six phylogenetic groups because the full-genome nucleotide sequences were used in the previous publication, apart from the method and model of tree construction.

Regarding the entropy analysis, multiple sequence alignment of the *Cap* gene revealed a high polymorphism of nucleic and amino acid sequences with 211 and 101 entropy peaks, respectively. Notably, the highest entropy value at amino acid position 149 implies high variability of this amino acid position, where the amino acid was detected under the diversifying selection by MEME method and found to be a part of linear (Table 4; Fig. 5) and discontinuous B-cell epitopes (Fig. 6 C). These findings suggest that certain positions within the *Cap* gene and Cap amino acid sequences exhibit high variability, which is a result of diversifying selection induced by host immunity, as observed in Porcine circovirus type 2 (PCV2) study [18].

The NLS is a short peptide that plays an important role in the transportation of proteins from the cytoplasm into the nucleus. In this study, the NLS was identified in the CanineCV Cap protein, and most of the amino acids were arginine. The existence of NLS in the Cap protein has been reported in PCV2 and PCV3 [19, 20]. Notably, an arginine-rich region at the N-terminus of NLS was also described [21]. A subcellular localization study is needed to elucidate the biological and functional characteristics of NLS in canine host cells providing knowledge of the virus life cycle.

Considering the selection pressure acting on the *Cap* gene, the mean dN/dS was less than 1, indicating that the CanineCV *Cap* gene is evolving under purifying selection, consistent with prior studies on CanineCV [22], PCV2 [23, 24], and PCV3 [25]. Purifying selection is the selective removal of deleterious mutations. Too strong purifying selection leads to a genetic diversity reduction. Parvovirus B19, a human parvovirus, is another ssDNA virus in which the Cap protein also evolved under purifying selection. Preserving its biological function was the reason [26]. As to whether the CanineCV Cap biological functions are associated with receptor binding, virus entry, or virus assembly, we have proposed that the purifying selection pressure acting on the Cap protein maintains its virological activity. Functional identification might clarify this speculation. The only codon under diversifying selection, significantly supported by all methods, was at position 50. We found that this codon residue was located in the recognized B-cells epitopic regions (Supplementary Tables 3, 4, 6, 7 and Fig. 6C). Furthermore, a codon at position 24, supported by three methods, was identified in B-cell epitopes (Supplementary Tables 3, 4, 5, and 7), while the codons at position 103 and 111, supported by three methods, were detected in the T-cell epitopic regions (Table 6). The positive selection site on the antigenic epitope was also indicated in PCV research [23, 25]. Our result suggests that this diversifying selection in these codon positions was partly driven by host immunological response.

In this study, B-cell epitopes analyzed based on the CanineCV Cap peptide sequence with characteristic parameters including antigenicity, hydrophilicity, flexibility, accessibility and turns prediction provided four consensus peptide chains (Fig. 5). The antigenic potential of these four regions was emphasized by the predicted discontinuous B-cell epitopes from the ElliPro server (Fig. 6 C). For T-cell epitopes prediction, the NetMHCpan-4.1 server proposed eleven peptides of MHC-I binding sites of canine T-cells that can interact with DLA molecule of canine MHC. However, based on the VaxiJen antigenicity score, with the cut-off set at 0.5, as a result, the remaining six peptide sequences which are RTRPLIRYR (position 15–23), KMFHLRLRR (position 31–39), YVRAKWINW (position 101–109), SVIQGANMV (position 212–220), MVWNGLSISL (position 219–228) and, SAYITFKEF (position 254–262) had antigenic potential to simulate immune response (Table 6). Recently, a study of CanineCV using a bioinformatic approach suggested five epitopes for epitope-based vaccine development [17]. Noteworthy, two epitopes, YQHLPPFRF (position 86–94) and YIRAKWINW (position 101–109), are similar to our proposed T-cell epitopes. Recently, Kaushik and colleagues identified the sequence DPLQDRSSSRSFNM (position 163–176) as a T-cell epitope on the Cap protein [27], which shares a consensus region with our predicted T-cell epitopes (Table 6). Together with the outcomes regardimg B-cell epitopes, we found seven out of eleven sequences that shared consensus regions with the B-cell epitope sequences: KMFHLRLRR (position 31–39), HLPPFRFYK (position 88–96), FIYDPLQDR (position 160–168), SVIQGANMV (position 212–220), MVWNGLSISL (position 219–228), VQYDISAYI (position 249–257), and SAYITFKEF (position 254–262) (Table 6). The discovery of T-cell epitope regions in previous studies, similar to those identified in our research, along with the detection of B-cell epitopes within T-cell epitope regions, emphasizes the potential importance of these antigenic sites. In this investigation, we only analyzed epitope regions through *in silico* analysis of one Capsid protein sequence, leading to inherent limitations. Expanding analysis to more sequences and conducting in vivo experiments is crucial for stronger evidence of immune epitopes on the CanineCV Capsid protein.

## Conclusions

We characterized the CanineCV *Cap* genome using cloning technique. The virus has high genetic and amino acid variability. Our CanineCV strain is grouped within the 3rd clade. The *Cap* gene of CanineCV appears to be influenced by purifying natural selection. However, the presence of positive selection within the predicted B-cell and T-cell epitopes implies that virus evolution is partly driven by host immunity. Evolving of the virus under other factors requires further elucidation. Our study provides not only the CanineCV genetic and evolutionary fundamental knowledge but also a new insight into immune-related epitopes of the viral Cap protein that can be applied for the development of immunodiagnostic tools and vaccine strategies for CanineCV infection in the future.

## Materials and methods

### Ethics statement

This study was approved by the Institutional Animal Care and Use Committee (IACUC) (No. 2031014) and the Institutional Biosafety Committee (IBC) (No. 1931036) of Chulalongkorn University, Thailand.

### Sample collection and DNA extraction

The secretion samples were collected via nasal and oropharyngeal swabs from a one-year-old female Golden Retriever dog at the Small Animal Teaching Hospital, Faculty of Veterinary Science, Chulalongkorn University, Thailand. The samples were immersed in 1% (v/v) sterile phosphate-buffered saline (PBS) and immediately used for CanineCV DNA extraction using the Viral DNA/RNA extraction kit (Geneaid, Taiwan), following the manufacturer’s protocol. The concentration and purity of DNA samples were defined at the 260/280 ratio using a Nanodrop® Lite spectrophotometer (Thermo Scientific™, USA). The extracted samples were kept at −80 °C until further use.

### Amplification of CanineCV Cap gene

The CanineCV *Cap* gene was amplified by conventional PCR using specific primers designed with the 4 base sequences (underline) at the 5’ end of forward primer with an overhang sequence (GTGG) in a pET100/D-TOPO® vector (Invitrogen, USA) as shown in Table 7. The PCR reaction mixture was performed in a total volume of 20 μL, containing 50 ng of DNA template, 0.5 μM of each primer, 200 μM of each deoxynucleotide triphosphate (dNTPs), 1x phusion GC buffer, nuclease free water and 0.4 U Phusion® High-Fidelity DNA Polymerase (New England Biolabs, UK). The thermocycling was carried out in a thermal cycler (Eppendorf, Germany) with an initial denaturation at 98 °C for 30 s, followed by 35 cycles of 98 °C for 10 s, 63 °C for 20 s, 72 °C for 30 s, and 72 °C for 10 min, providing 813 bp length of the full *Cap* gene (Table 7). The positive control was retrieved from a previous publication [10], while non-template control (NTC) was used as a negative control. The PCR products were run on 1% (w/v) agarose gel electrophoresis, stained with SYBR green fluorescence dye, and visualized under a UV transilluminator. The positive samples were purified using a NucleoSpin Extract II (Macherey-Nagel, Germany) according to the manufacturer’s instruction, and then submitted for bidirectional Sanger’s sequencing (Macrogen, Korea).

**Table 7:** Primer pairs used for amplification and sequencing of capsid (*Cap*) gene for CanineCV detection

Primer name	Primer sequence (5’- 3’)	Size (bp)
CanineCV-F1928	*CACC﻿*ATGCGCGTACGTAG	813
CanineCV-R1116	TTACAACTGTCGACCAGTTTCA	

### Cloning and sequencing of the CanineCV Cap gene

To construct recombinant CanineCV *Cap* gene, the purified PCR product was ligated unidirectionally into pET100/D-TOPO® vector (Invitrogen, USA). The ligation product was transformed into chemically competent *Escherichia coli* TOP10 cells and plated on Luria-Bertani (LB) agar with ampicillin. Positive colonies were selected, cultured overnight, and the recombinant plasmids were extracted from bacterial cultures using a PureDireX Plasmid miniPREP Kit (Bio-helix, Taiwan) for sequencing.

### CanineCV Capsid sequence and in silico analysis

#### Phylogenetic analysis

A CanineCV *Cap* sequence from this study and other ninety-four *Cap* sequences retrieved from the GenBank database were edited, aligned with the ClustalW function, and translated into amino acid using Bioedit software v.7.2 [28]. MEGA 7.0 program [29] was used to analyze the *Cap* sequences similarity, nucleotide composition (A%, T%, C%, G%), and nucleotide substitution rates. Bayesian Evolutionary Analysis Sampling Trees (BEAST) v.1.10.4 was applied to analyze the sequences, with the tree built using the HKY + G + I as the best-fit substitution model identified by MEGA. The tree was estimated by 10,000,000 Markov chain Monte Carlo (MCMC) sampling method which 25% of sampled trees were discarded. The reconstructed phylogenetic tree was visualized using FigTree v.1.4.4. The reliability of the phylogeny was supported by posterior probability branch support values of the tree. In addition, a Bayesian Skyride plot was demonstrated using a coalescent Gaussian Markov random field (GMRF) Bayesian Skyride model with the time-aware smoothing option, in combination with a random local clock model to estimate the past population dynamic of CanineCV.

#### Entropy analysis

Entropy patterns of the *Cap* nucleotide and amino acid sequences were evaluated by Bioedit software package version v.7.2 [28].

#### Haplotype diversity

DnaSP software v.6.0 [30] was employed to examine the haplotype diversity (Dh), nucleotide diversity (π), and average number of nucleotide differences (*K*). Templeton, Crandall, and Sing (TCS) Network was then generated by Population Analysis with the Reticulate Trees (popART) program [31].

#### Nuclear localization signal prediction

The Nuclear Sorting signal prediction server (http://mleg.cse.sc.edu/seqNLS.) was used to identify the nuclear localization signal (NLS) region in the CanineCV Cap protein sequence. The submitted amino acid residues with the highest score (> 0.89) were predicted to be a part of NLS [32].

#### Evolutionary analysis

Selection pressure acting on the *Cap* gene was evaluated by a combination of four evolutionary analyses including Single-Likelihood Ancestor Counting (SLAC) [33], Fixed Effects Likelihood (FEL) [33], Fast Unconstrained Bayesian AppRoximation (FUBAR) [34], and Mixed Effects Model of Evolution (MEME) [35] to estimate diversifying or purifying natural selection using the Datamonkey online program [36] based on the ratios between nonsynonymous and synonymous substitution rates (dN/dS). The diversifying selection was accepted when the *p*-value ≤ 0.1 in SLAC, FEL, MEME, and the posterior probability ≥ 0.9 in FUBAR [37, 38]. Sites presented positive signal at least one method were interpreted as being under diversifying selection pressure [38].

#### B-cell and T-cell epitopes analysis

To identify the potential antigenic regions for B-cell, the translated Cap amino acid sequence was submitted to the Immune Epitope Database and Analysis Resource (IEDB-AR) (http://www.iedb.org). The prediction of continuous B-cell epitopes was computed based on physiochemical properties of the protein sequences using the Kolaskar & Tongaonkar antigenicity, BepiPred linear epitope, Parker hydrophilicity, Emini surface accessibility, Chou & Fasman Beta-turn prediction, and Karplus & Schulz flexibility prediction. Then, ElliPro online program was used to establish the 3D structures of antigens for the prediction of the discontinuous B-cell epitopes. Additionally, *in silico* T-cell epitope evaluation was done using the T-cell epitopes MHC Binding method in IEDB Analysis Resource on NetMHCpan-4.1 server to predict epitopes on the Cap protein sequence to a specific MHC class I molecule (MHC-I). The MHC-I epitope binding sites with a strong binding ability was considered when the percentage rank was lower than 0.5% [39]. The predicted epitope sequences were evaluated for antigenicity using the VaxiJen server v.2.0 to determine their potential as antigens. Epitopes with a Vaxijen score greater than 0.5 were considered to be suitable antigens [40].

### Electronic supplementary material

Below is the link to the electronic supplementary material.

Supplementary Material 1

Supplementary Material 2

Supplementary Material 3

Supplementary Material 4

Supplementary Material 5

Supplementary Material 6

Supplementary Material 7

## Data Availability

All the data supporting our findings is contained within the manuscript. A full-length coding CanineCV *Cap* sequence has been deposited in NCBI GenBank under accession ON863358.

## References

[1] Kapoor A, Dubovi EJ, Henriquez-Rivera JA, Lipkin WI (2012). Complete genome sequence of the first canine circovirus. J Virol.

[2] Anderson A, Hartmann K, Leutenegger CM, Proksch AL, Mueller RS, Unterer S (2017). Role of canine circovirus in dogs with acute haemorrhagic diarrhoea. Vet Rec.

[3] Cruz TF, Batista TN, Vieira EM, Portela LMF, Baccarin AM, Gradiz JJ et al. Genomic characterization of canine circovirus detected in a dog with intermittent hemorrhagic gastroenteritis in Brazil. Ciência Rural. 2020;50(5).

[4] Decaro N, Martella V, Desario C, Lanave G, Circella E, Cavalli A (2014). Genomic characterization of a circovirus associated with fatal hemorrhagic enteritis in dog, Italy. PLoS ONE.

[5] Dowgier G, Lorusso E, Decaro N, Desario C, Mari V, Lucente MS (2017). A molecular survey for selected viral enteropathogens revealed a limited role of canine circovirus in the development of canine acute gastroenteritis. Vet Microbiol.

[6] Hsu HS, Lin TH, Wu HY, Lin LS, Chung CS, Chiou MT (2016). High detection rate of dog circovirus in diarrheal dogs. BMC Vet Res.

[7] Li L, McGraw S, Zhu K, Leutenegger CM, Marks SL, Kubiski S (2013). Circovirus in tissues of dogs with vasculitis and hemorrhage. Emerg Infect Dis.

[8] Tuong NM, Piewbang C, Rungsipipat A, Techangamsuwan S (2021). Detection and molecular characterization of two canine circovirus genotypes co-circulating in Vietnam. Vet Q.

[9] Altan E, Seguin MA, Leutenegger CM, Phan TG, Deng X, Delwart E (2019). Nasal virome of dogs with respiratory infection signs include novel taupapillomaviruses. Virus Genes.

[10] Piewbang C, Jo WK, Puff C, van der Vries E, Kesdangsakonwut S, Rungsipipat A (2018). Novel canine circovirus strains from Thailand: evidence for genetic recombination. Sci Rep.

[11] Dankaona W, Mongkholdej E, Satthathum C, Piewbang C, Techangamsuwan S (2022). Epidemiology, genetic diversity, and association of canine circovirus infection in dogs with respiratory disease. Sci Rep.

[12] Thaiwong T, Wise AG, Maes RK, Mullaney T, Kiupel M (2016). Canine circovirus 1 (CaCV-1) and Canine Parvovirus 2 (CPV-2): recurrent dual infections in a papillon breeding colony. Vet Pathol.

[13] Van Kruiningen HJ, Heishima M, Kerr KM, Garmendia AE, Helal Z, Smyth JA (2019). Canine circoviral hemorrhagic enteritis in a dog in Connecticut. J Vet Diagn Invest.

[14] Beikpour F, Ndiana LA, Sazmand A, Capozza P, Nemati F, Pellegrini F et al. Detection and genomic characterization of canine circovirus in Iran. Animals. 2022;12(4). 10.3390/ani12040507 PMC8868521 35203215

[15] Blanchard P, Mahe D, Cariolet R, Keranflec’h A, Baudouard MA, Cordioli P (2003). Protection of swine against post-weaning multisystemic wasting syndrome (PMWS) by porcine circovirus type 2 (PCV2) proteins. Vaccine.

[16] Wang Z, Shi Y, Wang Y, Zhao L, Cui X, Wen S (2020). Detection of Antibodies against Canine Circovirus in naturally and experimentally infected canines by recombinant capsid enzyme-linked immunosorbent assay. Front Vet Sci.

[17] Jain P, Joshi A, Akhtar N, Krishnan S, Kaushik V (2021). An immunoinformatics study: designing multivalent T-cell epitope vaccine against canine circovirus. J Genet Eng Biotechnol.

[18] Correa-Fiz F, Franzo G, Llorens A, Segalés J, Kekarainen T (2018). Porcine circovirus 2 (PCV-2) genetic variability under natural infection scenario reveals a complex network of viral quasispecies. Sci Rep.

[19] Hou Q, Hou S, Chen Q, Jia H, Xin T, Jiang Y (2018). Nuclear localization signal regulates porcine circovirus type 2 capsid protein nuclear export through phosphorylation. Virus Res.

[20] Mou C, Wang M, Pan S, Chen Z. Identification of nuclear localization signals in the ORF2 protein of porcine circovirus type 3. Viruses. 2019;11(12). 10.3390/v11121086 PMC6950156 31766638

[21] Liu BY, Gao B, Liu MZ, Zhang TT, Liu BS, Chen ZL (2020). High repetitive arginine in the anterior of PCV3 capsid protein is a severe obstacle for its expression in E. Coli. AMB Express.

[22] Giraldo-Ramirez S, Rendon-Marin S, Vargas-Bermudez DS, Jaime J, Ruiz-Saenz J (2020). First detection and full genomic analysis of Canine Circovirus in CPV-2 infected dogs in Colombia, South America. Sci Rep.

[23] Franzo G, Cortey M, Segales J, Hughes J, Drigo M (2016). Phylodynamic analysis of porcine circovirus type 2 reveals global waves of emerging genotypes and the circulation of recombinant forms. Mol Phylogenet Evol.

[24] Wei R, Xie J, Theuns S, Nauwynck HJ (2019). Changes on the viral capsid surface during the evolution of porcine circovirus type 2 (PCV2) from 2009 till 2018 may lead to a better receptor binding. Virus Evol.

[25] Chen Y, Xu Q, Chen H, Luo X, Wu Q, Tan C et al. Evolution and genetic diversity of porcine circovirus 3 in China. Viruses. 2019;11(9). 10.3390/v11090786 PMC6783837 31461875

[26] Stamenkovic GG, Cirkovic VS, Siljic MM, Blagojevic JV, Knezevic AM, Joksic ID (2016). Substitution rate and natural selection in parvovirus B19. Sci Rep.

[27] Kaushik V, Jain P, Akhtar N, Joshi A, Gupta LR, Grewal RK (2022). Immunoinformatics-aided design and in vivo validation of a peptide-based Multiepitope Vaccine Targeting Canine Circovirus. ACS Pharmacol Translational Sci.

[28] Hall TA, editor. BioEdit: a user-friendly biological sequence alignment editor and analysis program for Windows 95/98/NT. Nucleic acids symposium series; 1999: [London]: Information Retrieval Ltd., c1979-c2000.

[29] Kumar S, Stecher G, Tamura K (2016). MEGA7: Molecular Evolutionary Genetics Analysis Version 7.0 for bigger datasets. Mol Biol Evol.

[30] Rozas J, Ferrer-Mata A, Sánchez-DelBarrio JC, Guirao-Rico S, Librado P, Ramos-Onsins SE, et al. DnaSP 6: DNA sequence polymorphism analysis of large data sets. Mol Biol Evol. 2017;34(12):3299–302. 10.1093/molbev/msx248 29029172

[31] Leigh JW, Bryant D (2015). Popart: full-feature software for haplotype network construction. Methods Ecol Evol.

[32] Lin JR, Hu J (2013). SeqNLS: nuclear localization signal prediction based on frequent pattern mining and linear motif scoring. PLoS ONE.

[33] Kosakovsky Pond SL, Frost SDW (2005). Not so different after all: a comparison of methods for detecting amino Acid sites under selection. Mol Biol Evol.

[34] Murrell B, Moola S, Mabona A, Weighill T, Sheward D, Kosakovsky Pond SL (2013). FUBAR: a fast, unconstrained Bayesian AppRoximation for Inferring Selection. Mol Biol Evol.

[35] Murrell B, Wertheim JO, Moola S, Weighill T, Scheffler K, Kosakovsky Pond SL (2012). Detecting Individual sites subject to episodic diversifying selection. PLoS Genet.

[36] Weaver S, Shank SD, Spielman SJ, Li M, Muse SV, Kosakovsky Pond SL (2018). Datamonkey 2.0: a modern web application for characterizing selective and other evolutionary processes. Mol Biol Evol.

[37] Zheng G, Lu Q, Wang F, Xing G, Feng H, Jin Q (2020). Phylogenetic analysis of porcine circovirus type 2 (PCV2) between 2015 and 2018 in Henan Province, China. BMC Vet Res.

[38] Velazquez-Salinas L, Zarate S, Eberl S, Gladue DP, Novella I, Borca MV. Positive selection of ORF1ab, ORF3a, and ORF8 genes drives the early evolutionary trends of SARS-CoV-2 during the 2020 COVID-19 pandemic. Front Microbiol. 2020;11. 10.3389/fmicb.2020.550674 PMC7644918 33193132

[39] Reynisson B, Alvarez B, Paul S, Peters B, Nielsen M (2020). NetMHCpan-4.1 and NetMHCIIpan-4.0: improved predictions of MHC antigen presentation by concurrent motif deconvolution and integration of MS MHC eluted ligand data. Nucleic Acids Res.

[40] Doytchinova IA, Flower DR (2007). VaxiJen: a server for prediction of protective antigens, tumour antigens and subunit vaccines. BMC Bioinformatics.

