# Supplementary material for: Molecular characterization of canine circovirus based on the Capsid gene in Thailand
Source: BMC Vet Res. 2024 Jul 13;20:312. doi: 10.1186/s12917-024-04120-w (PMC11245861; doi:10.1186/s12917-024-04120-w)
Supplement: Supplementary file 2 — Supplementary Material 2 [file 12917_2024_4120_MOESM2_ESM.docx]

**Supplementary Table 2** Codon positions under purifying selection

| Method | Codon position | dN/dS | *p*-value |
| --- | --- | --- | --- |
| FEL | 16 | 0.411 | 0.097 |
|  | 20 | 0.110 | 0.000 |
|  | 67 | 0.068 | 0.091 |
|  | 85 | 0.093 | 0.008 |
|  | 102 | 0.206 | 0.015 |
|  | 134 | 0.081 | 0.024 |
|  | 148 | 0.373 | 0.039 |
|  | 189 | 0.021 | 0.022 |
|  | 198 | 0.263 | 0.078 |
|  | 215 | 0.066 | 0.018 |
| SLAC | 20 | 0.181 | 0.001 |
|  | 85 | 0.134 | 0.020 |
|  | 134 | 0.083 | 0.032 |
|  | 189 | 0.070 | 0.081 |
| FUBAR | 20 | 0.169 | 0.988 |
|  | 85 | 0.131 | 0.974 |
|  | 102 | 0.240 | 0.950 |
|  | 134 | 0.110 | 0.916 |
|  | 148 | 0.391 | 0.967 |
|  | 215 | 0.061 | 0.959 |

The significant threshold was set to *p*-value ≤ 0.1 in SLAC and FEL methods, and to posterior probability ≥ 0.9 in FUBAR method.
