# Supplementary material for: Molecular characterization of canine circovirus based on the Capsid gene in Thailand
Source: BMC Vet Res. 2024 Jul 13;20:312. doi: 10.1186/s12917-024-04120-w (PMC11245861; doi:10.1186/s12917-024-04120-w)
Supplement: Supplementary file 3 — Supplementary Material 3 [file 12917_2024_4120_MOESM3_ESM.docx]

**Supplementary Table 3** Result of Bepipred Linear Epitope Prediction 2.0

| No. | Start | End | Peptide | Length |
| --- | --- | --- | --- | --- |
| 1 | 5 | 30 | RHARASRRRYRTRPLIRYRRRRQNNF | 26 |
| 2 | 42 | 63 | TADWPTAPVKPTNDPQTETPLL | 22 |
| 3 | 77 | 94 | LQASHGTGDFQHLPPFRF | 18 |
| 4 | 103 | 117 | RAKWINWPKTMMENV | 15 |
| 5 | 127 | 181 | GEDQGRGNATRSHLDPGTVPGLSEPPKDPNKAPFIYDPLQDRSSSRSFNMASGFK | 55 |
| 6 | 189 | 206 | MFTQDITSPSATAPWLTR | 18 |
| 7 | 231 | 245 | IKDMRPTTPDTTTSQ | 15 |
| 8 | 263 | 267 | DYETG | 5 |

Average = 0.531, Maximum = 0.675, Minimum = 0.270, Threshold = 0.5
