# Supplementary material for: Molecular characterization of canine circovirus based on the Capsid gene in Thailand
Source: BMC Vet Res. 2024 Jul 13;20:312. doi: 10.1186/s12917-024-04120-w (PMC11245861; doi:10.1186/s12917-024-04120-w)
Supplement: Supplementary file 4 — Supplementary Material 4 [file 12917_2024_4120_MOESM4_ESM.docx]

**Supplementary Table 4** Result of Parker Hydrophilicity Prediction

| No. | Start | End | Peptide | Length |
| --- | --- | --- | --- | --- |
| 1 | 5 | 15 | RHARASRRRYR | 11 |
| 2 | 23 | 29 | RRRRQNN | 7 |
| 3 | 49 | 59 | PVKPTNDPQTE | 11 |
| 4 | 80 | 85 | SHGTGD | 6 |
| 5 | 123 | 157 | LDLDGEDQGGNATRSHLDPGTVPGLSEPPKDPNK | 34 |
| 6 | 165 | 176 | LQDRSSSRSFNM | 12 |
| 7 | 193 | 199 | DITSPSA | 7 |
| 8 | 230 | 247 | QIKDMRPTTPDTTTSQIP | 18 |
| 9 | 263 | 267 | DYETG | 5 |

Average = 1.574, Maximum = 7.057, Minimum = -3.329, Threshold = 1.574
