# Supplementary material for: Molecular characterization of canine circovirus based on the Capsid gene in Thailand
Source: BMC Vet Res. 2024 Jul 13;20:312. doi: 10.1186/s12917-024-04120-w (PMC11245861; doi:10.1186/s12917-024-04120-w)
Supplement: Supplementary file 5 — Supplementary Material 5 [file 12917_2024_4120_MOESM5_ESM.docx]

**Supplementary Table 5** Result of Emini Surface Accessibility prediction

| No. | Start | End | Peptide | Length |
| --- | --- | --- | --- | --- |
| 1 | 3 | 16 | VRRHARASRRRYRT | 14 |
| 2 | 21 | 29 | RYRRRRQNN | 9 |
| 3 | 51 | 59 | KPTNDPQTE | 9 |
| 4 | 150 | 156 | EPPKDPN | 7 |
| 5 | 164 | 170 | PLQDRSS | 7 |
| 6 | 231 | 242 | IKDMRPTTPDTT | 12 |

Average = 1.000, Maximum = 6.682, Minimum = 0.124, Threshold = 1.000
