# Supplementary material for: Molecular characterization of canine circovirus based on the Capsid gene in Thailand
Source: BMC Vet Res. 2024 Jul 13;20:312. doi: 10.1186/s12917-024-04120-w (PMC11245861; doi:10.1186/s12917-024-04120-w)
Supplement: Supplementary file 6 — Supplementary Material 6 [file 12917_2024_4120_MOESM6_ESM.docx]

**Supplementary Table 6** Result of Chou & Fasman Beta-Turn Prediction

| No. | Start | End | Peptide | Length |
| --- | --- | --- | --- | --- |
| 1 | 25 | 29 | RRQNN | 5 |
| 2 | 45 | 59 | WPTAPVKPTNDPQTE | 15 |
| 3 | 81 | 88 | HGTGDFQH | 8 |
| 4 | 108 | 111 | NWPK | 4 |
| 5 | 126 | 158 | DGEDQGRGNATRSHLDPGTVPGLSEPPKDPNKA | 33 |
| 6 | 164 | 176 | PLQDRSSSRSFNM | 13 |
| 7 | 180 | 186 | FKRGLTP | 7 |
| 8 | 194 | 200 | ITSPSAT | 7 |
| 9 | 204 | 210 | LTRGTPW | 7 |
| 10 | 219 | 225 | MVWNGLS | 7 |
| 11 | 235 | 245 | RPTTPDTTTSQ | 11 |
| 12 | 264 | 266 | YET | 3 |

Average = 1.023, Maximum = 1.371, Minimum = 0.747, Threshold = 1.023
