# Supplementary material for: Molecular characterization of canine circovirus based on the Capsid gene in Thailand
Source: BMC Vet Res. 2024 Jul 13;20:312. doi: 10.1186/s12917-024-04120-w (PMC11245861; doi:10.1186/s12917-024-04120-w)
Supplement: Supplementary file 7 — Supplementary Material 7 [file 12917_2024_4120_MOESM7_ESM.docx]

**Supplementary Table** 7 Result of Karplus & Schulz Flexibility Prediction

| No. | Start | End | Peptide | Length |
| --- | --- | --- | --- | --- |
| 1 | 10 | 18 | SRRRYRTRP | 9 |
| 2 | 24 | 29 | RRRQNN | 6 |
| 3 | 46 | 61 | PTAPVKPTNDPQTETP | 16 |
| 4 | 82 | 85 | GTGD | 4 |
| 5 | 125 | 158 | LDGEDQGRGNATRSHLDPGTVPGLSEPPKDPNKA | 34 |
| 6 | 164 | 173 | PLQDRSSSRS | 10 |
| 7 | 180 | 188 | FKRGLTPKP | 9 |
| 8 | 191 | 199 | TQDITSPSA | 9 |
| 9 | 205 | 209 | TRGTP | 5 |
| 10 | 230 | 247 | QIKDMRPTTPDTTTSQIP | 18 |

Average = 1.007, Maximum = 1.120, Minimum = 0.907, Threshold = 1.007
